# Clark’s Nutcrackers (*Nucifraga columbiana*) Flexibly Adapt Caching Behavior to a Cooperative Context

**DOI:** 10.3389/fpsyg.2016.01643

**Published:** 2016-10-25

**Authors:** Dawson Clary, Debbie M. Kelly

**Affiliations:** Department of Psychology, University of Manitoba, WinnipegMB, Canada

**Keywords:** cache protection, Clark’s nutcrackers, complex cognition, cooperation, corvid

## Abstract

Corvids recognize when their caches are at risk of being stolen by others and have developed strategies to protect these caches from pilferage. For instance, Clark’s nutcrackers will suppress the number of caches they make if being observed by a potential thief. However, cache protection has most often been studied using competitive contexts, so it is unclear whether corvids can adjust their caching in beneficial ways to accommodate non-competitive situations. Therefore, we examined whether Clark’s nutcrackers, a non-social corvid, would flexibly adapt their caching behaviors to a cooperative context. To do so, birds were given a caching task during which caches made by one individual were reciprocally exchanged for the caches of a partner bird over repeated trials. In this scenario, if caching behaviors can be flexibly deployed, then the birds should recognize the cooperative nature of the task and maintain or increase caching levels over time. However, if cache protection strategies are applied independent of social context and simply in response to cache theft, then cache suppression should occur. In the current experiment, we found that the birds maintained caching throughout the experiment. We report that males increased caching in response to a manipulation in which caches were artificially added, suggesting the birds could adapt to the cooperative nature of the task. Additionally, we show that caching decisions were not solely due to motivational factors, instead showing an additional influence attributed to the behavior of the partner bird.

## Introduction

Why individuals work for the interests of others, at times even against their own benefit, has long been a source of intrigue for evolutionary biologists and comparative psychologists (Axelrod and Hamilton, 1981). Such cooperative behaviors are now known to be evolutionarily feasible if they provide inclusive fitness benefits (Brosnan and Bshary, 2010; Lehmann and Rousset, 2010). However, the conditions in which an individual chooses to act in a cooperative fashion are still unclear (Bshary and Oliveira, 2015). In primates for instance, although many cooperative acts have been explained by kin selection (Richerson and Boyd, 2005), some instances of cooperation are between non-kin and seem to have more direct and proximate benefits (e.g., alliance formation: Watts, 2002; Gilby et al., 2009; group hunting: Boesch and Boesch, 1989; Boesch, 1994; Watts and Mitani, 2002; cooperation experiments: Hauser et al., 2003; Greenberg et al., 2010; Hare and Kwetuenda, 2010; Horner et al., 2011; Melis et al., 2011).

The decision-making behind whether to act cooperatively can require complex cognitive abilities, such as cost benefit analysis and social cognition. Acting cooperatively can come with immediate risks, such as in third party conflict resolution (Silk, 1992; Watts, 2002); not acting cooperatively can prevent one from benefiting from joint ventures, such as group hunting (Boesch, 1994), or cause one to be socially ostracized (Boehm, 1999). Thus, it would be advantageous for an individual to be capable of judging whether the cooperative act will come at a cost or benefit, and adjust the degree to which one cooperates accordingly (Trivers, 1971). The *social competence hypothesis* (Taborsky and Oliveira, 2012; Bshary and Oliveira, 2015) argues that social behaviors evolved to be flexible and that individuals can select from their full range of behavioral options to adjust to ever changing contexts. However, whether non-human animals have these requisite cognitive skills to allow for this cognitive flexibility is still being questioned (Stevens and Hauser, 2004; Brosnan et al., 2010).

Complex cognition has, historically, been thought to be predominantly limited to primates (Humphrey, 1976). Indeed, primates have been shown capable of some of the cognitive abilities thought to underlie cooperation, such as adjusting to cost/benefit changes (Marsh and MacDonald, 2012), or responding to the mental states of others (e.g., Hare et al., 2001; Flombaum and Santos, 2005). Thus, primates have been the primary source of research examining psychological aspects of non-human animal cooperation. Some evidence suggests chimpanzees can be flexible in how they cooperate if allowed to negotiate with a partner (Melis et al., 2009) and if cooperation is more lucrative than completing tasks alone (Bullinger et al., 2011; though cooperation is not always achieved by chimpanzees, see Melis et al., 2008; Brosnan et al., 2009). Furthermore, other primates, such as capuchin monkeys, show socially dependent cooperation by adjusting rates of cooperation depending on the partner’s cooperative effort (de Waal and Berger, 2000; Brosnan et al., 2006; Takimoto and Fujita, 2011) and the degree of competition (de Waal and Davis, 2003). Therefore, prompting an individual to consider and respond to the needs of potential partners could be an important precursor for successful cooperation (Trivers, 1971; Eisenberg and Mussen, 1989; Silk, 2007; Burkart et al., 2009; de Waal and Suchak, 2010).

Corvids are increasingly reported to be clever problem solvers, making them another promising animal for the study of cooperative decision-making. Research investigating corvids has shown their cognitive abilities rival those of primates, especially in the domain of social cognition (Emery and Clayton, 2004, 2009). As expressed by their food caching (i.e., hiding food for later use) behaviors, corvids seem to consider the visual perspective of other birds when hiding caches (Dally et al., 2004; Clayton et al., 2007; Bugnyar, 2011), infer the intent of other birds to steal their caches (Emery et al., 2004; Bugnyar and Heinrich, 2006; Clary and Kelly, 2011; Shaw and Clayton, 2012) and use their own experience as thieves to anticipate the need to protect their caches (Emery and Clayton, 2001). From these findings, corvids seem to have a striking aptitude for complex social cognition; however, most of these impressive behaviors have been reported in the context of competitive interactions. Therefore, it is unclear whether the caching behaviors of corvids are rigid species-typical responses, or whether these behaviors can be flexibly adjusted to accommodate cooperative contexts. Corvids have been found to make cooperative decisions in both natural ecological (cooperative breeding: Woolfenden and Fitzpatrick, 1996; reciprocal agonistic support: Fraser and Bugnyar, 2011; food sharing: de Kort et al., 2006; von Bayern et al., 2007; Scheid et al., 2008) and non-caching laboratory experiments (Stephens et al., 2002; Scheid and Noë, 2010; Schwab et al., 2012; Ostojić et al., 2013). For example, rooks will cooperate with a partner to complete a string-pulling task during which two birds must jointly pull on opposite ends of a string to move a platform containing food within reach (Scheid and Noë, 2010). As another example, male Eurasian jays will anticipate the food preferences of their mate; provisioning the female with food she has not been sated with (Ostojić et al., 2013). So although some studies have investigated corvid cooperation, the cooperative caching potential of corvids (and cognitive flexibility required for such a behavior) has yet to be examined.

During this study, we developed a novel procedure to examine whether Clark’s nutcrackers (*Nucifraga columbiana*), which are relatively non-social compared to other corvids (e.g., crows, magpies, pinyon jays, Western scrub jays), use a competitive or cooperative caching strategy if given a cooperative caching task. Unlike more social species, nutcrackers only form small, temporary flocks, aggressively defend their territories, and prefer to eat and cache away from others (Tomback, 1998). When examined in a competitive context, nutcrackers suppress caching when observed by a conspecific (Tomback, 1998; Clary and Kelly, 2011; in press), whereas relatively more social Western scrub-jays have been found to increase caching (Emery et al., 2004). During these competitive experiments, birds were allowed to cache either alone or observed by a conspecific. After being observed caching, some of the bird’s caches were pilfered by the observer (in view of the original caching bird), before the original caching bird was allowed to retrieve the remaining caches in private. Clary and Kelly (2011) speculated that caching behaviors might be influenced by sociality, with less social species viewing caching situations as more competitive than social species, and less prone to accept risk to their caches, accounting for the opposing responses of nutcrackers and scrub jays in this task. Hence, the current study examined whether nutcrackers are capable of overcoming their typical, and well-established, tendency to suppress caching in the presence of conspecifics if the experimental contingencies make it beneficial to do so, or if their caching behaviors are constrained by species-typical responses devoid of flexible social cognitive control. Thus, nutcrackers were given a caching task in which birds made caches that were only recovered by a partner, and in turn they only received their partner’s caches. The birds experienced this situation over repeated trials, and thus had the opportunity to learn the partner’s caching patterns. Therefore, if the nutcrackers flexibly adjust their caching strategies they should respond to the cooperative context by continuing to make caches that will be delivered to the partner bird. Alternatively, if the nutcrackers’ caching behavior is constrained by species-specific tendencies (i.e., always suppress caching when observed), then a decrease in caching over time would be expected, despite each bird gaining reinforcement by recovering the partner’s caches.

## Materials and Methods

### Subjects

Fourteen sexually mature, wild-caught Clark’s nutcrackers (7 female, 7 male) were used in this experiment. As in the competitive caching task previously used with nutcrackers (Clary and Kelly, 2011), each bird was tested with an opposite sex partner for the duration of the experiment. The nutcrackers were of unknown age, but had been in the laboratory for a minimum of 7 years. The birds had participated in a pilot study (unpublished data) in which they cached in the presence of a conspecific, but none had experienced cache theft in an experimental context. The colony rooms were maintained at a stable temperature of 22°C and a 12 h light cycle, with light onset at 0.700. Birds were housed in individual cages (48 cm length × 48 cm width × 73 cm height) with multiple perches for the duration of the experiment with water and grit provided *ad libitum*. All birds were fed *ad libitum* except on test days (see Procedure). Nutcrackers were fed a diet consisting of turkey starter, parrot pellets, sunflower seeds, mealworms, peanuts, pine nuts, and a vitamin supplement. All animal care procedures were approved by the University of Manitoba Animal Care Committee (approval #F10-029) and are in accordance with the guidelines of the Canadian Council on Animal Care.

### Apparatus

Birds were tested in their home cages, which were transported to an experimental room. The cages were placed on a table (121 cm long × 60 cm wide) and positioned so, when perched, the birds faced one another. The table was surrounded by white curtains (200 cm long × 175 cm wide) to provide a uniform viewing environment. An ice cube tray filled with sand was provided to each bird to allow caching (tray consisted of 26 wells arranged in a 13 × 2 matrix; overall dimensions: 49.5 cm long × 11 cm wide). A combination of Mega Building Blocks^TM^ (i.e., plastic toy blocks of varying shape, size, and color) affixed to the trays uniquely identified each bird’s caching tray, a procedure commonly (and successfully) used to facilitate tray discrimination (e.g., Emery et al., 2004; Clary and Kelly, 2011). All trials were recorded using four EverFocus 1/3″ color digital cameras and the software package BiObserve.

### Procedure

#### Food Deprivation

All birds were food deprived 24 h prior to participating in a weekly trial. A weekly trial consisted of a *Caching Session* during the first day and a *Retrieval Session* during the second day. During the *Caching Session* birds had the opportunity to eat and/or cache 50 pine nuts. After the *Caching Session*, birds were supplemented with a restricted amount of regular feed to maintain the bird at a healthy weight. After completing the *Retrieval Session*, birds were returned to an *ad libitum* diet until the next week’s trial. Birds were weighed daily to ensure a healthy weight was maintained throughout the experiment and to measure both motivation to eat when outside the weekly trials and motivation to cache during each *Caching Session*.

### Baseline Trials

Each bird experienced six Baseline trials. Before the trial, both birds of a pair were transported to the experiment room. During the *Caching Session*, an experimenter gave the first bird of the pair its visually unique caching tray, placed in the center of the cage and positioned horizontally relative to the partner bird’s viewing position, and a dish containing 50 pine nuts. The tray’s central position allowed for the entire tray to be easily viewable by the partner bird. The 50 pine nuts allowed for the bird to eat to satiety, as well as cache freely, while having a surplus at the end of the trial. The first bird was allowed to eat and cache the pine nuts for 45 min, after which, the tray and pine nut dish was removed from the bird’s cage and placed visibly between the two cages, out of reach of both birds. The second bird of the pair was then given its visually unique caching tray and dish of 50 pine nuts and allowed to eat and cache for 45 min. The bird that cached first was alternated on a weekly basis. After the *Caching Session* the birds were returned to the colony room and the number of pine nuts eaten, the number of caches made in the tray, and the number of caches made external to the tray, for each bird, was documented.

The next day, the birds were returned to the experiment room to participate in the *Retrieval Session*. During the *Retrieval Session*, both birds were provided with their original caching tray and allowed to recover their cached food (i.e., both birds recovered their caches at the same time). *Retrieval Sessions* lasted 1 h, after which, the birds were returned to the colony room and the number of remaining caches documented. Nutcrackers have previously been shown to remember caches accurately after 285 days (Balda and Kamil, 1992); therefore, if caches remained in the tray, then subsequent *Retrieval Sessions* were conducted on the following day until all caches were recovered. This was done to prevent memories of previously made and unrecovered caches from interfering with the caches made during the next *Caching Session*.

#### Cache Sharing

Each bird experienced 12 Cache Sharing trials, starting the week immediately following the completion of Baseline trials. Procedures for these trials were identical to those of the Baseline trials, with the exception that during the *Retrieval Session*, instead of the birds receiving their own caching tray, the birds received their partner’s caching tray.

#### Cache Addition

Each bird experienced six Cache Addition trials following completion of all Cache Sharing trials. Procedures were identical to the Cache Sharing condition except that after the *Caching Session*, the experimenter added caches to the trays so that each individual received twice the maximum number of caches received on a single trial from the partner during Cache Sharing. Therefore, upon completion of *Retrieval Sessions*, total pine nut consumption for each bird far exceeded the amount consumed during the Baseline and Cache Sharing conditions. The caches were distributed evenly across all of the tray’s wells. Note, these caches were added by the experimenter after the birds were returned to the colony room, and therefore, the birds never saw the caches being added to the tray. This procedure was conducted to examine whether an exaggerated cooperative response would be elicited from the birds if it seemed that the partner had become more generous.

### Statistical Analysis

The data were blocked so as to compare the average of the six Baseline trials, the first six Cache Sharing trials, the second six Cache Sharing trials, and the six Cache Addition trials. As the measured behaviors were highly variable according to individual and sex, the variables (number of caches remaining in the tray, number of external caches, number of pine nuts eaten, number of caching events, and weight of the bird as measured on the morning prior to each *Caching Session*) were standardized by computing them as a proportion of the values measured during the Baseline trials. Caching events (herein referred to simply as ‘events’ to disambiguate from caches remaining in the tray) were scored from the videos and defined as any instance when the bird placed a pine nut in the sand of the tray, and therefore included all re-caches (i.e., repeated placements of a single pine nut), serving as a more general measure of caching activity. Latency from the time the birds jumped down to the bottom of the cage to when they made contact with the tray was also scored from the recorded trials. Due to a program error, some trials were not recorded properly and could not be scored (Baseline: 4/84; Cache Sharing 1: 2/84; Cache Sharing 2: 17/84; Cache Addition: 2/84). This imbalance was accounted for with our use of a mixed-effect modeling technique (described below), which is robust to missing values (Baraldi and Enders, 2010).

To assess if any of the variables changed over different blocks or trials of the experiment, we used linear mixed-effects models with block/trial and sex inputted as fixed effects along with their interaction, and subject inputted as a random effect. We also created linear mixed-effects models based on the Cache Sharing trials alone to assess whether the birds’ caching was influenced by social factors or by motivational factors. This model included weight of the focal bird, the number of caches made by the partner, and the number of caching events of the partner inputted as fixed effects, and subject inputted as a random effect. We excluded the first trial of the Cache Sharing block as the birds would not have had a chance to learn the procedures by this point, and replaced this trial with the first Cache Addition trial. Since we alternated which bird cached first, one bird of the pair on any given trial would not have the opportunity to base their caching on the partner’s caching, unless relying on what they experienced during the previous week’s trial. To account for this we computed a running mean of two trials for the number of caches made by the focal bird, so that each data point represented the average of when a bird went first and second. For all analyses parameter estimation was achieved using residual maximum likelihood and degrees of freedom were estimated using Satterthwaite approximation. Analyses were performed in R version 3.1.2 using the *lme4* (Bates et al., 2014) and *multcomp* packages (Hothorn et al., 2008).

## Results

### Changes Over Blocks/Trials

#### Caching

**FIGURE 1:**
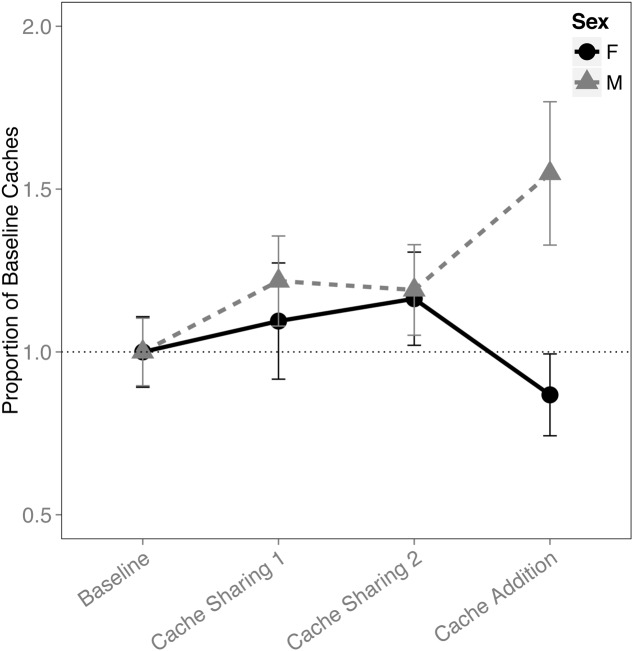
**Proportion of baseline caches made (±SEM) during each block of six trials.** Caching amounts (number of caches left in the tray at the end of each trial) during Cache Sharing and Cache Addition conditions were computed as proportions relative to the caching amounts measured during Baseline. Therefore, Baseline values are 1.0 by definition, and for the remaining conditions, values above 1.0 indicate an increase in caching relative to Baseline, and values below 1.0 indicate a decrease in caching relative to Baseline.

**FIGURE 2:**
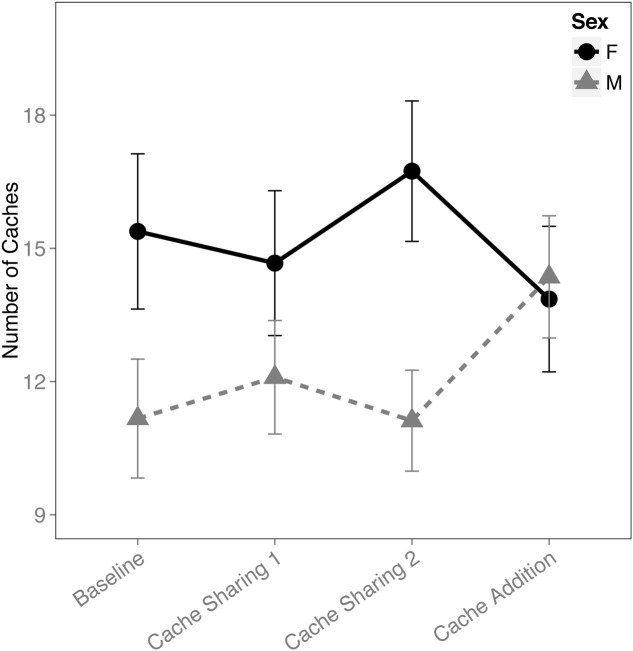
**Mean number of caches made (±SEM) during each block of six trials**.

When examining the proportion of caches made in the tray(s), there was an interaction between block and sex (*F*_(3,316)_ = 2.833, *p* = 0.038). This was due to an increase in proportion of caches made by the males during the Cache Addition block compared to Baseline (*z* = 2.889, *p* = 0.026, *d* = 1.09; **Figure 1**), whereas no detectable statistical change was observed for females during Cache Addition relative to other blocks (Baseline: *z* = 0.695, *p* = 0.979; Cache Sharing 1: *z* = 1.194, *p* = 0.761; Cache Sharing 2: *z* = 1.556, *p* = 0.500). The change in proportion of caches made eliminated the tendency for females to make more absolute number of caches than males (see **Figure 2**).

**FIGURE 3:**
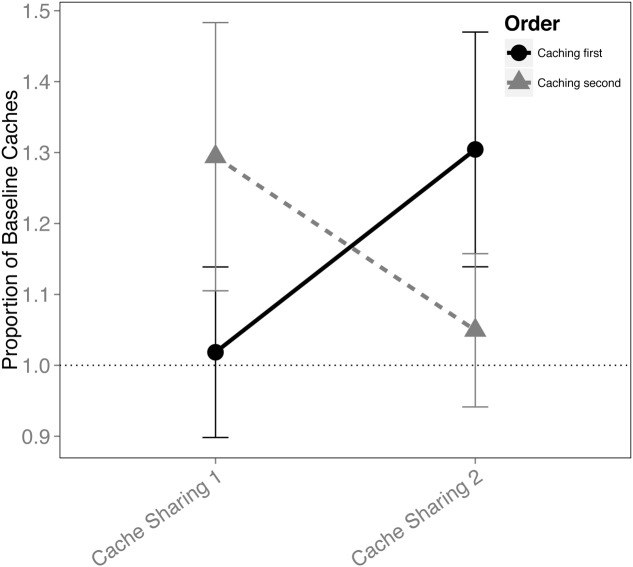
**Proportion of baseline caches made (±SEM) during the first and second half of Cache Sharing trials separated by whether the birds cached before or after their partner.** Caching amounts (number of caches left in the tray at the end of each trial) during Cache Sharing and Cache Addition conditions were computed as proportions relative to the caching amounts measured during Baseline. Therefore, values above 1.0 indicate an increase in caching relative to Baseline, and values below 1.0 indicate a decrease in caching relative to Baseline.

When the order in which the birds cached was analyzed for the Cache Sharing blocks (i.e., only Cache Sharing 1 and Cache Sharing 2), there was an interaction between the order of caching and each block of Cache Sharing (*F*_(1,148)_ = 4.221, *p* = 0.042), but no interaction between Cache Sharing block and sex (*F*_(1,148)_ = 0.138, *p* = 0.711), and no three way interaction between order, Cache Sharing block, and sex (*F*_(1,148)_ = 1.716, *p* = 0.192). That is, the effect of order of caching was different (reversed) between Cache Sharing 1 and Cache Sharing 2 (**Figure 3**). Both males and females reduced caching in the later trials (Cache Sharing 2) when they were the second bird to cache, but increased caching when they were the first bird to cache.

**FIGURE 4:**
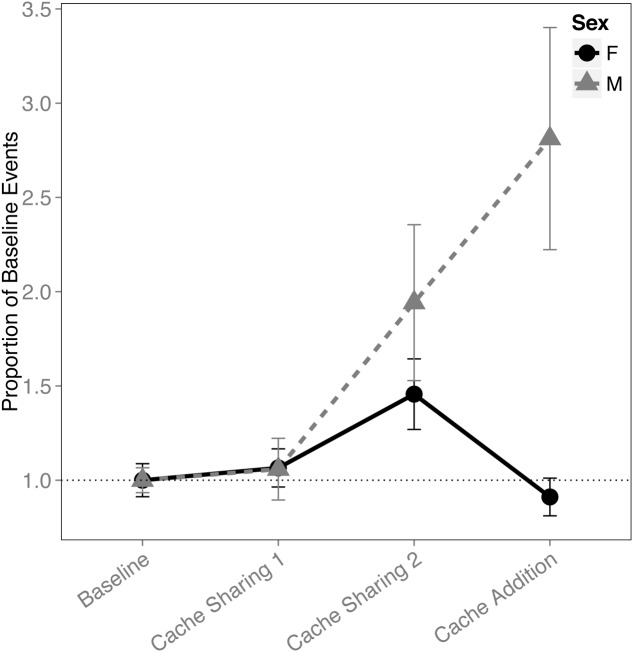
**Proportion of baseline events (±SEM) during each block of six trials.** Events (a more general measure of caching activity combining caches and re-caches) during Cache Sharing and Cache Addition conditions were computed as proportions relative to the events measured during Baseline. Therefore, Baseline values are 1.0 by definition, and for the remaining conditions, values above 1.0 indicate an increase in events relative to Baseline, and values below 1.0 indicate a decrease in events relative to Baseline.

When analyzing the proportion of events (i.e., combined caches and re-caches) across all four blocks, there was an interaction between block and sex (*F*_(3,291)_ = 9.110, *p* < 0.001). This was driven by an increase in proportion of events made by males (Cache Sharing 1 – Cache Sharing 2: *z* = 2.688, *p* = 0.047, *d* = 1.01; Cache Sharing 2 – Cache Addition: *z* = 2.800, *p* = 0.034, *d* = 1.06; **Figure 4**), whereas the females’ proportion of events returned to Baseline levels.

For the proportion of external caches we found no effect of block (*F*_(3,316)_ = 0.207, *p* = 0.892), sex (*F*_(1,12)_ = 0.086, *p* = 0.775), nor a block by sex interaction (*F*_(3,316)_ = 1.781, *p* = 0.151).

#### Latency

**FIGURE 5:**
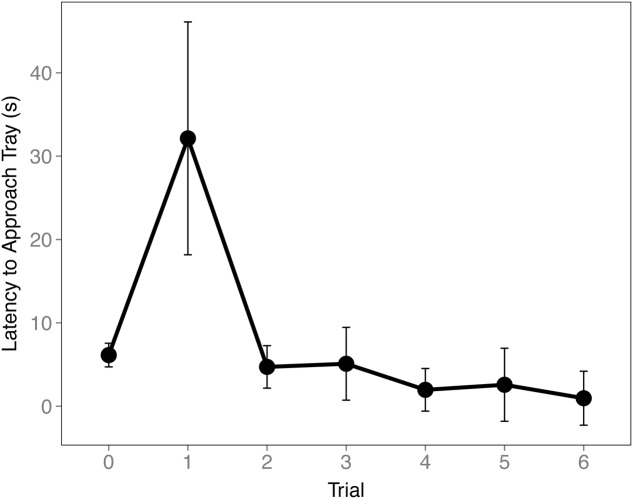
**Mean latency to approach tray (±SEM) during the final Baseline trial (0) and the first six Cache Sharing trials.** Latency was measured from when the bird first landed on the bottom of the cage to when the bird first made contact with the caching tray.

To ensure the birds understood the trays were switched during the Cache Sharing trials we examined their latency to approach the tray. An increase in latency to approach the tray was found on the first Cache Sharing trial compared to the last Baseline trial (*z* = 3.281, *p* = 0.006, *d* = 0.80; **Figure 5**) suggesting the birds recognized a difference in trays. Latency decreased during subsequent trials suggesting the birds quickly came to expect the new tray.

#### Eating/Weight

**FIGURE 6:**
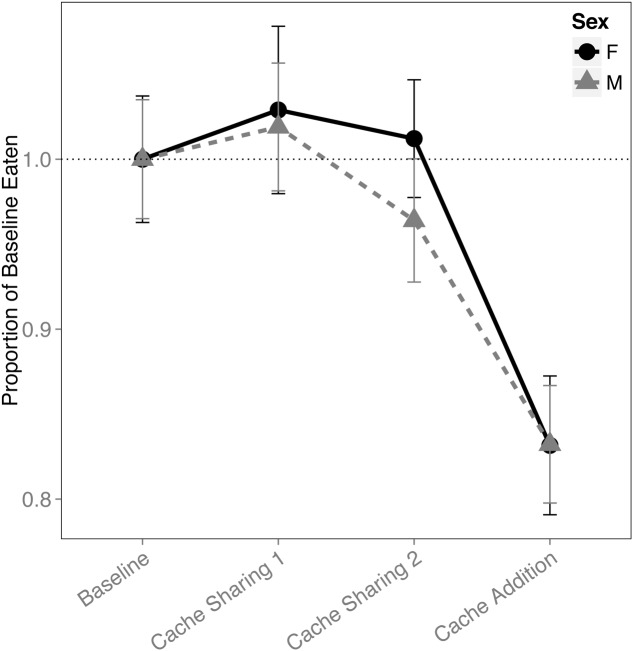
**Proportion of baseline pine nuts eaten (±SEM) during each block of six trials.** Number of pine nuts eaten (during *Caching Sessions*) during Cache Sharing and Cache Addition conditions were computed as proportions relative to the pine nuts eaten during Baseline. Therefore, Baseline values are 1.0 by definition, and for the remaining conditions, values above 1.0 indicate an increase in pine nuts eaten relative to Baseline, and values below 1.0 indicate a decrease in pine nuts eaten relative to Baseline.

There was a main effect of block on the proportion of pine nuts eaten (*F*_(3,316)_ = 12.445, *p* < 0.001). Both males and females ate proportionally fewer pine nuts during *Caching Sessions* of the Cache Addition block compared to all other blocks (Baseline: *z* = 4.819, *p* < 0.001, *d* = 1.29; Cache Sharing 1: *z* = 5.506, *p* < 0.001, *d* = 1.47; Cache Sharing 2: *z* = 4.476, *p* < 0.001, *d* = 1.20; **Figure 6**).

**FIGURE 7:**
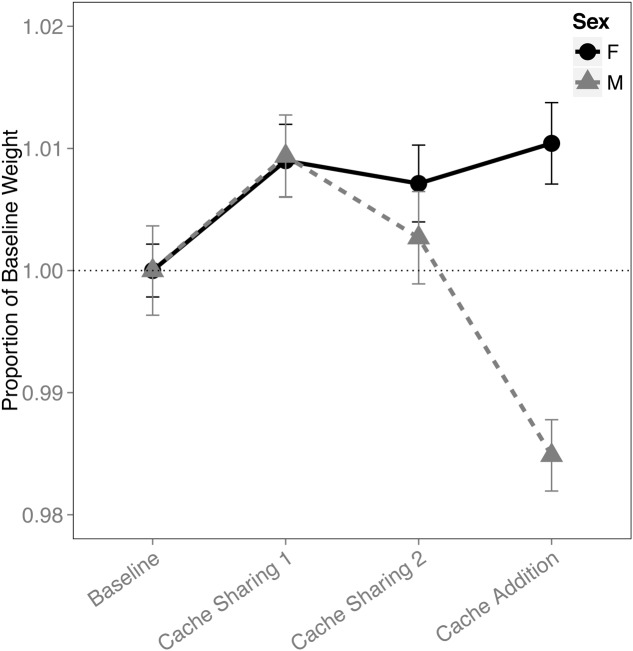
**Proportion of baseline weight (±SEM) during each block of six trials.** Weight of the bird (as measured on the morning of each *Caching Session*) during Cache Sharing and Cache Addition conditions were computed as proportions relative to the weight of each bird measured during Baseline (as measured on the morning of each Baseline *Caching Session*). Therefore, Baseline values are 1.0 by definition, and for the remaining conditions, values above 1.0 indicate an increase in weight relative to Baseline, and values below 1.0 indicate a decrease in weight relative to Baseline.

An interaction of block and sex was found for the proportion of the birds’ weight (*F*_(3,316)_ = 8.696, *p* < 0.001). This was due to a reduction in the males’ weight during the Cache Addition block compared to all other blocks (Baseline: *z* = 3.631, *p* = 0.002, *d* = 1.37; Cache Sharing 1: *z* = 5.878, *p* < 0.001, *d* = 2.22; Cache Sharing 2: *z* = 4.275, *p* < 0.001, *d* = 1.62; **Figure 7**), whereas females showed a marginal increase in weight during Cache Addition relative to Baseline (*z* = 2.498, *p* = 0.075, *d* = 0.94), though this weight was not different from the Cache Sharing blocks (Cache Sharing 1: *z* = 0.340, *p* = 1.000; Cache Sharing 2: *z* = 0.789, *p* = 0.957).

### Predictors of Caching Behavior

#### Males

When examining proportion of caches there was no effect of the partner’s caches (*R* = -0.147, *F*_(1,75.05)_ = 3.390, *p* = 0.070) nor weight of the focal bird (*R* = -2.256, *F*_(1,79.97)_ = 0.394, *p* = 0.532), but there was a marginal positive effect of the partner’s events (*R* = 0.151, *F*_(1,77.72)_ = 3.871, *p* = 0.053). When examining proportion of events, there was a positive effect of the partner’s events (*R* = 0.606, *F*_(1,78.94)_ = 8.495, *p* = 0.005) and a negative effect of weight of the focal bird (*R* = -33.173, *F*_(1,78.97)_ = 11.810, *p* = 0.001), but no effect of the partner’s tray caches (*R* = -0.058, *F*_(1,75.35)_ = 0.0706 *p* = 0.791).

#### Females

When examining proportion of caches, we found no effect of the partner’s tray caches (*R* = -0.088, *F*_(1,35.87)_ = 0.282, *p* = 0.600), the partner’s events (*R* = -0.043, *F*_(1,47.70)_ = 0.351, *p* = 0.557), nor the weight of the focal bird (*R* = 11.378, *F*_(1,52.67)_ = 2.816, *p* = 0.100). When examining proportion of events, there was a positive effect of both the partner’s tray caches (*R* = 0.288, *F*_(1,53.05)_ = 4.866, *p* = 0.032) and the partner’s events (*R* = 0.122, *F*_(1,69.15)_ = 4.835, *p* = 0.031), but no effect of the focal bird’s weight (*R* = 6.309, *F*_(1,71.34)_ = 1.476, *p* = 0.228).

## Discussion

The birds maintained caching levels throughout the Cache Sharing trials, despite recognizing the trays were switched, showing no detectable change in strategy: neither an increase in caching indicative of overt cooperation, nor a decrease in caching indicative of competition. Though this finding may reflect the birds’ baseline motivational drive to cache (Clayton and Dickinson, 1999), it is in contrast to previous findings that nutcrackers suppress their caching over time in response to witnessing cache theft in a competitive context (Clary and Kelly, 2011; in press). Furthermore, the birds tended to cache more when they were the first bird of the pair to cache, suggesting the birds may have attempted to prevent defection from the partner by appearing more cooperative (though reduced caching was still observed when a bird was the second of a pair to cache). The lack of a more pronounced reciprocal response from the bird caching second may have precluded more apparent cooperation from being detected during Cache Sharing trials.

When the birds experienced artificially exaggerated cooperation during Cache Addition, the males seemed to respond cooperatively, whereas the females maintained baseline levels of caching behaviors. Interestingly, the change in caching reduced the inequity in absolute number of pine nuts exchanged between the partners. The increase in caching by males during the Cache Addition block is peculiar in that these caches only benefit the other individual. By this point in the experiment the birds would have had ample opportunity to learn they were never provided with an opportunity to recover their own caches, yet the males responded to receiving more caches by caching more themselves and incurring additional costs by eating fewer of the 50 allotted pine nuts during *Caching Sessions*. Therefore, if non-social corvids, like nutcrackers, do have an inherent tendency to cache competitively as suggested by Clary and Kelly (2011), then this tendency was easily over-ridden to fit the cooperation-biased structure and non-ecologically relevant Cache Sharing aspects of this caching task.

Another interpretation could be that the nutcrackers’ caching was already suppressed during Baseline, despite extended experience learning their caches were always returned in an intact state from the Baseline trials and the earlier conducted pilot study. This learning may have interfered with detecting further cache suppression. Looking at the absolute caching values in comparison with our previous research (Clary and Kelly, 2011) indicates this may have been the case, though mainly for the males. Under this interpretation, the males’ responses during Cache Addition would be an alleviation of existing cache suppression. Regardless of interpretation, the results indicate the males adjusted their caching decisions based on the experimental context.

There were also changes in motivational variables during the Cache Addition block, as the increase in caching by males corresponded with a decrease in weight and pine nuts eaten during the *Caching Session*, whereas females, along with maintaining caching behaviors, maintained their weight and showed a reduction in pine nuts eaten. The reduction in pine nuts eaten by the birds occurred even though the surplus of pine nuts given to them did not require a reduction to accommodate increased caching, and the lengthy trial times (45 min) did not restrict their behavioral choices. Thus, it seems the birds anticipated receiving caches during the *Retrieval Session* of Cache Addition trials. This was shown by the birds eating less during the *Caching Session*, as well as by the males no longer compensating for the weekly food deprivation by consuming more food during the 5 days between weekly trials, shown by their decreased weight as measured on the morning prior to each bird’s *Caching Session*. The reduction in weight was unlikely to be due to other factors, such as stress, as we would expect such a stress response to emerge much earlier if the birds found the experiment aversive, rather than corresponding with the Cache Addition condition, at which point the birds should be most accustomed to the experimental procedures. Therefore, although it is possible the birds did not understand that trials would be repeated, these results suggest the birds came to expect receiving food in future trials or sessions.

As predictors of caching behavior, a combination of motivational and social factors were found to influence the birds’ caching decisions, with both weight of the bird, but more consistently, the events of the partner explaining variance in the focal bird’s caching behaviors. Therefore, the birds seemed to match the overall caching activity (caching and re-caching) of the other bird. Overall caching activity (events) was likely a more viable way for an observing bird to judge the behavior of the caching bird, as accounting for each cache would require constant attention throughout the 45 min trial and full visual access to the cache locations, which could be obstructed based on the caching bird’s body position. These results were likely driven by re-caches, possibly indicating the birds were either still engaging in cache protection or attempting to extract more cooperation from the partner by appearing more cooperative (though less likely considering evidence for such deception is rare amongst animals). Importantly however, the relationship between the overall caching activities of the pairs shows the birds were not responding solely based on motivational factors, but also attending to the behavior of their partner, consistent with previous research indicating that nutcrackers use observational spatial memory to pilfer the caches of others (Bednekoff and Balda, 1996).

From an ecological perspective, considering there was an initial investment that is only recouped through a similar investment by the partner, the nutcrackers’ behavior could be labeled as reciprocity (Raihani and Bshary, 2011). That cooperation emerged in this experimental structure is interesting, as past research has found that animals often converge on mutual defection during similar tasks (Flood et al., 1983; Clements and Stephens, 1995; Hall, 2003). However, our experimental structure contained features that game theoretical models predict to facilitate cooperation: iteration and alternation (Doebeli and Hauert, 2005). Indeed, when conditions and payoffs are carefully constructed to favor cooperation, experimental contexts find cooperation can emerge (Rutte and Taborsky, 2007; St. Pierre et al., 2009; Viana et al., 2010; Pinto et al., 2011).

Another interpretation could be that the increase in caching was due to courtship behavior, accounting for the more pronounced response of the males, and would not require the birds to understand the cooperative aspects of the task (though still cooperative in an ecological sense: Stevens and Gilby, 2004). For many species, males provision females to facilitate pair bonding or mating attempts (Lewis and South, 2012). Though both males and females were cooperative in that they continued to cache at baseline levels throughout the Cache Sharing trials instead of suppressing their caching (as is normal for this species), it is curious that only males responded so strongly to Cache Addition. It could be that males were advertising their quality to the females by caching more and eating less. However, if this behavior was related to courtship it is odd that it only manifested during the Cache Addition block (18 weeks after experiment onset), during which the males also benefited by receiving extra caches. Furthermore, courtship displays were not observed during the trials of the current study, nor among the male-female pairs used by Clary and Kelly (2011), which resulted in cache suppression.

The results could also be due to some other ecological characteristic that differentiates male and female caching behaviors. Unfortunately, field studies on this aspect of caching behaviors by Clark’s nutcrackers are lacking, with no reports examining differences in caching or pilfering rates between males and females. We have not detected any sex differences for caching rates in competitive experimental contexts (Clary and Kelly, 2011; in press), nor has there been any observations to indicate that nutcrackers may tolerate pilferage of their caches by mates, as has been reported for pinyon jays (Balda, 2002). Clark’s nutcrackers do exhibit extensive bi-parental care, with both the males and females incubating eggs and provisioning hatched young (Tomback, 1998), which would undermine explanations based on parental investment.

Although we could describe the behavior of the nutcrackers as cooperative from an ecological perspective, this need not be the case psychologically. The results could be due to an associative mechanism. By adding caches during Cache Addition, it could be argued that positive associations with the environment and the behaviors engaged in within that environment are enhanced. If receiving caches acts as a positive reinforcer for caching behavior, then it would be expected that the frequency of caching behaviors would increase. Indeed, the number of events increased over trials, although only for the males. That females made slightly more caches than males, and thus experienced a net cache loss during the exchange in Cache Sharing, could explain why females did not show an increase in tray caches or events before Cache Addition – but not after. It is unclear why females would be resistant to this associative mechanism when it should be strongest. Additionally, the ‘predictors of caching behavior’ analyses revealed that caching decisions were not largely driven by the caches received (reinforcement based), but instead influenced mostly by the activity level of the partner. Similarly, previous research using greater trial numbers has found no positive feedback due to cache recovery (Thom and Clayton, 2014; Clary and Kelly, in press), despite corvids typically learning rapidly in caching tasks (trials 1-3: e.g., Correia et al., 2007; Raby et al., 2007; Clary and Kelly, 2011). Furthermore, when caching behavior has been explicitly tested with respect to associative learning, nutcrackers did not alter their caching behavior when cache loss was paired with an inanimate object (Clary and Kelly, 2011).

Independent of which explanations are invoked, this type of response to unexpected caches could provide a mechanism for offsetting the high pilferage rates in the wild as noted by Vander Wall and Jenkins (2003). Ostensibly, caching is a disadvantageous strategy prone to being infiltrated by cheating strategies; however, the authors showed that caching could be maintained as an evolutionarily advantageous strategy through reciprocity of pilferage, though this reciprocity was based on exploitation, rather than altruism. If individuals respond to finding unexpected caches by increasing their own caching, as shown here, it would offset the costs of the theft victim by making it more likely they will reciprocally pilfer the caches of others in the future. Furthermore, if natural caching exchanges resemble cooperation in a game theoretical sense, then we may expect decisions on where to cache to be influenced by similar factors identified in this literature (e.g., Doebeli and Hauert, 2005), particularly the spatial distribution and stability of neighboring individuals allowing for iteration and choice of partners.

Our novel task provides a variety of strengths over previous tasks devised to investigate cooperative behaviors in non-human animals. First, due to the caching context, the food is not visible at the time of cooperation. During studies with primates, visible food has been found to inhibit prosocial choices (Silk et al., 2005; Jensen et al., 2006; Vonk et al., 2008; Bullinger et al., 2011). Second, researchers have expressed concern over some species’ tendency to discount the future in favor of current motivational needs (Stephens et al., 2002). Using Clark’s nutcrackers, a caching species that relies on long-term spatial memory to recover food stores (see Kamil and Balda, 1990 for a review), minimizes this concern as they likely make caching decisions to satisfy future, rather than immediate, motivational needs (as is the case for Western scrub jays: Correia et al., 2007; Raby et al., 2007; Thom and Clayton, 2014). Third, during experiments with greater physical distance between animals, null results for cooperation have been found (Jensen et al., 2006; Vonk et al., 2008). During our experiment there was small physical separation between the two birds, which has been argued to facilitate cooperation (Horner et al., 2011).

From a proximate perspective, it seems the nutcrackers were willing to cooperate – as long as that cooperation was established and maintained (see also St. Pierre et al., 2009), as males first required cache addition before increasing their own caching. In this sense, the males were willing to work against their own interests, only if they could accrue some selfish benefit. From an ultimate perspective, this research suggests that high levels of sociality (group living) are not required for the development of flexible social cognition, nor the ability to respond adaptively to cooperative contexts, and consistent with observations that social learning mechanisms are not unique to social species (Heyes, 2012). Conversely, game theoretical models predict that smaller group sizes favor the development of cooperation (Doebeli and Hauert, 2005). It may then be that the conditions for successful cooperation favor moderately social species, especially those with the cognitive abilities to track the more dynamic social aspects of cooperation, such as individual characteristics (e.g., dominance, reputation) and intentions. Here, using a novel task, we show that a relatively non-social species, which typically suppresses caching in the presence of others (Clary and Kelly, 2011) and prefers to make and retrieve caches solitarily (Tomback, 1998), can show flexibility in their caching strategy according to changing social contexts. This behavioral flexibility is consistent with the social competence hypothesis that suggests phenotypic plasticity allows animals to cope with their social world and predicts animals to optimize their social choices to account for changing contextual factors (Bshary and Oliveira, 2015). The novel procedure described here generates exciting new questions regarding the flexibility of corvid caching and further testing is certainly required to evaluate how animals acquire the ability to reason about social partners, to cooperate based on those judgments, and to isolate the mechanisms responsible for cooperation.

## Author Contributions

DC and DK developed the study; DC conducted the experiment and analyzed the data. DC wrote the majority of manuscript with comments and input from DK. Both DC and DK approved the final version of the manuscript and agree to be accountable for all aspects of the work.

## Conflict of Interest Statement

The authors declare that the research was conducted in the absence of any commercial or financial relationships that could be construed as a potential conflict of interest.The reviewer SB and the handling Editor declared their shared affiliation, and the handling Editor states that the process nevertheless met the standards of a fair and objective review.
